# ID 267 - Implementação do Núcleo de Avaliação de Tecnologias em Saúde: um marco para qualificação das decisões na gestão da Secretaria de Saúde do Estado da Bahia

**DOI:** 10.5327/2237-9622.2025.v34s1.267

**Published:** 2025-11-25

**Authors:** Carolina Santos Silva, Bárbara de Castro Santos Silva, Gleidson Cardoso Spínola, Manuela Fernandes de Almeida Mello, Rouseli Gonçalves de Menezes

**Keywords:** Avaliação de Tecnologias em Saúde, Secretaria Estadual de Saúde, processo de trabalho, tomada de decisão

## Abstract

**Introdução:**

Nos últimos anos, a área da saúde tem testemunhado uma rápida evolução tecnológica com novas oportunidades e desafios para a gestão de sistemas de saúde em todo o mundo. Para esse enfrentamento, a Secretaria de Saúde do Estado da Bahia (Sesab) implantou o Núcleo de Avaliação de Tecnologias em Saúde (Nats) como estratégia de preencher uma lacuna na avaliação e na incorporação de tecnologias, respondendo às necessidades específicas do sistema de saúde na perspectiva local. Este trabalho tem como objetivo descrever a experiência da criação do Nats/Sesab, destacando a sua importância e os resultados advindos com sua instituição.

**Método:**

A implantação foi iniciada em agosto de 2022 com distintos e simultâneos movimentos, tais quais: descrição da implantação de um Nats no programa de governo, como estratégia para apoiar as decisões da gestão e a consolidação da assistência farmacêutica; formação de uma equipe para compor o núcleo; apoio do Projeto ATS Educação – ProadiSUS, apresentado pelo Conselho Nacional de Secretários de Saúde (Conass) para qualificação dos profissionais na Avaliação de Tecnologias em Saúde (ATS); elaboração de documentos iniciais de regimento interno e do fluxo para análise de proposta de ATS; e um diagnóstico das instituições geridas pela Sesab, realizado pela Diretoria de Ciência, Tecnologia e Inovação em Saúde (Ditec), por meio de questionário eletrônico, objetivando identificar profissionais interessados em aplicar e promover a ATS no Sistema Único de Saúde (SUS) na Bahia. Ainda em 2022, a Rede Brasileira de Avaliação de Tecnologias em Saúde (Rebrats) deferiu a solicitação de filiação do Nats/Sesab. Em 2023, após as eleições estaduais, foi publicada a portaria que instituiu o Nats, bem como seu regimento interno e o fluxo para análise de proposta de incorporação (FAP-ATS). A assunção de inúmeras demandas em ATS favoreceu a ampliação da equipe.

**Resultados:**

Desde então, o Nats/Sesab vem realizando atividades de apoio à tomada de decisão dos gestores com elaboração de produtos de ATS, aprimorando a capacidade regulatória do Estado na incorporação de tecnologias. Em fase final, o Nats está a elaborar dois protocolos estaduais, um referente a uma nova tecnologia e um acerca da ampliação do uso de outra, ambos desencadeados após estudos de ATS realizados. A qualificação interna dos membros tem acontecido com os cursos e as oficinas ofertados pelo Hospital Moinho de Ventos, Hospital Alemão Oswaldo Cruz e Rebrats. Para o público externo, o Nats realizou seu primeiro seminário de ATS, com 123 participantes presenciais, além de estar adquirindo uma base científica de dados para as instituições de saúde do Estado, que está em fase avançada para disponibilização de curso de especialização em ATS. A divulgação do núcleo e dos resultados dos estudos tem ocorrido por meio da participação em eventos científicos, incluindo a apresentação de trabalhos.

**Conclusão:**

O Nats/Sesab fornece recomendações baseadas em evidências científicas relacionadas à adoção de tecnologias, bem como apoia gestores e profissionais de saúde na tomada de decisão, contribuindo para a sustentabilidade do sistema. A implantação do Nats representa um passo significativo para respostas referentes às necessidades emergentes, para a melhoria contínua dos serviços de saúde e para a promoção de uma gestão mais eficiente da saúde pública.

